# Supplementary material for: Dynamic Expression of EpCAM in Primary and Metastatic Lung Cancer Is Controlled by Both Genetic and Epigenetic Mechanisms
Source: Cancers (Basel). 2022 Aug 25;14(17):4121. doi: 10.3390/cancers14174121 (PMC9454530; doi:10.3390/cancers14174121)
Supplement: Supplementary file 1 [file cancers-14-04121-s001.zip › cancers-1833448-supplementary.pdf]

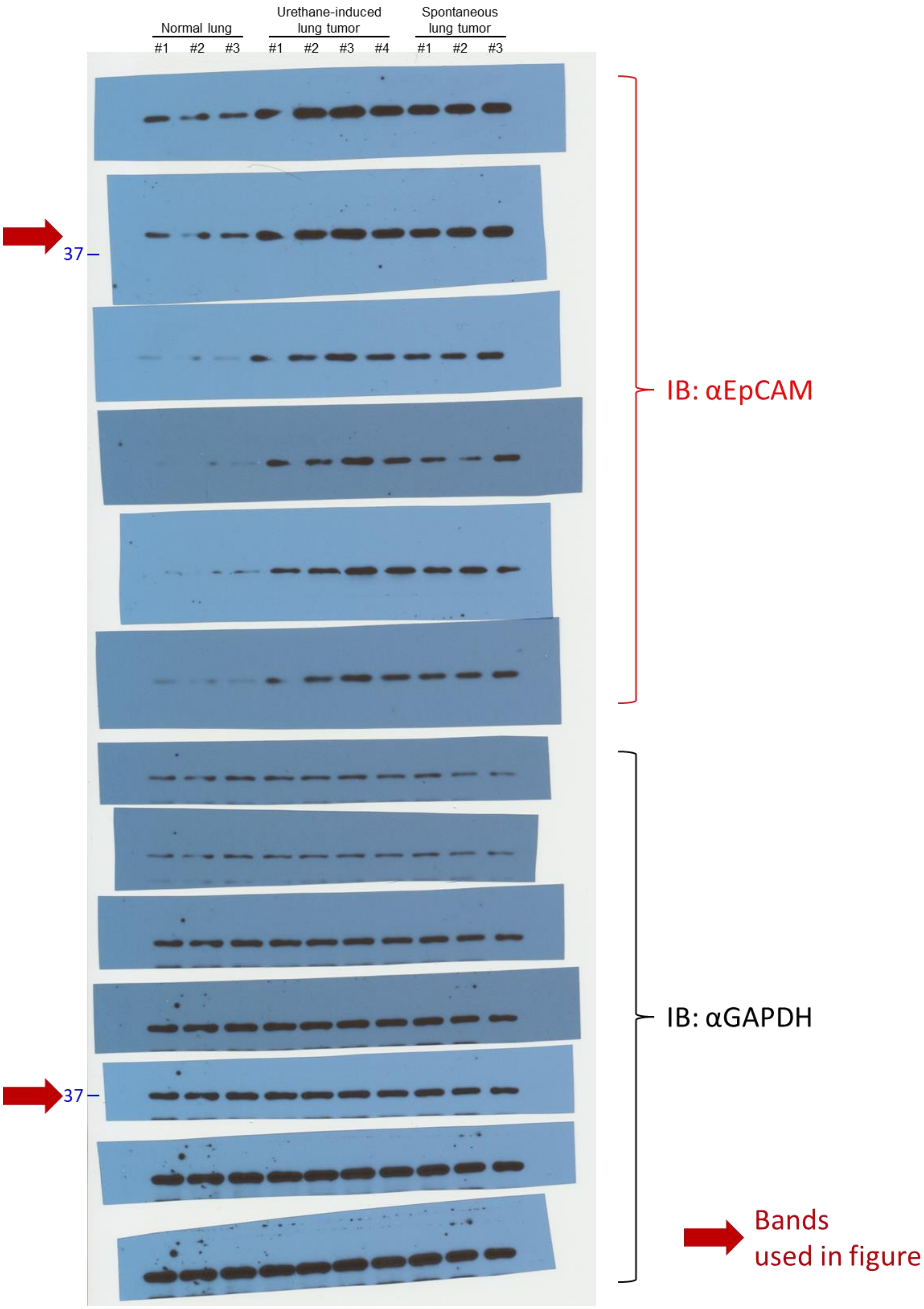


**Figure S1. Original western blot used in Figure 1I.**

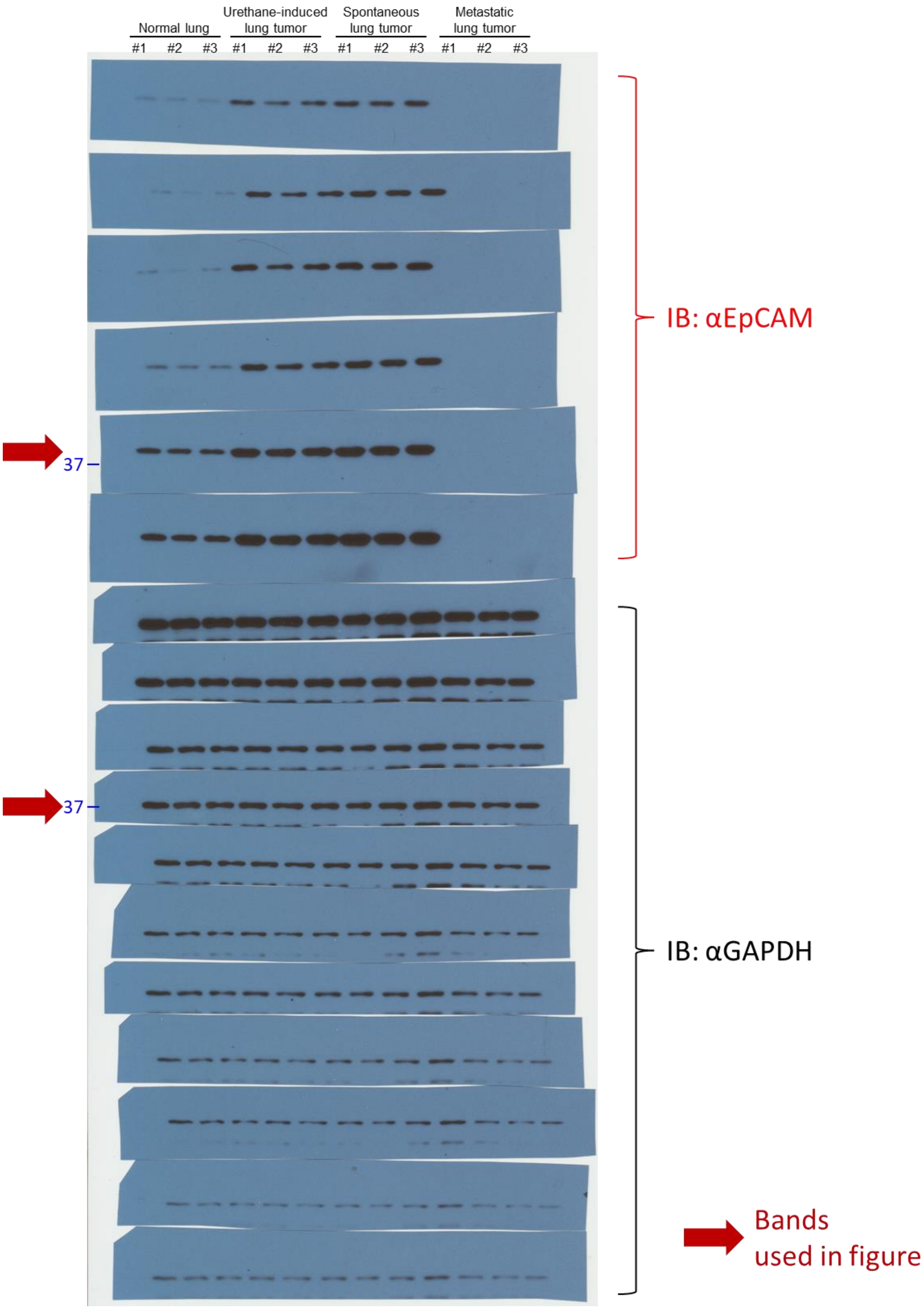


**Figure S2. Original western blot used in Figure 4D.**

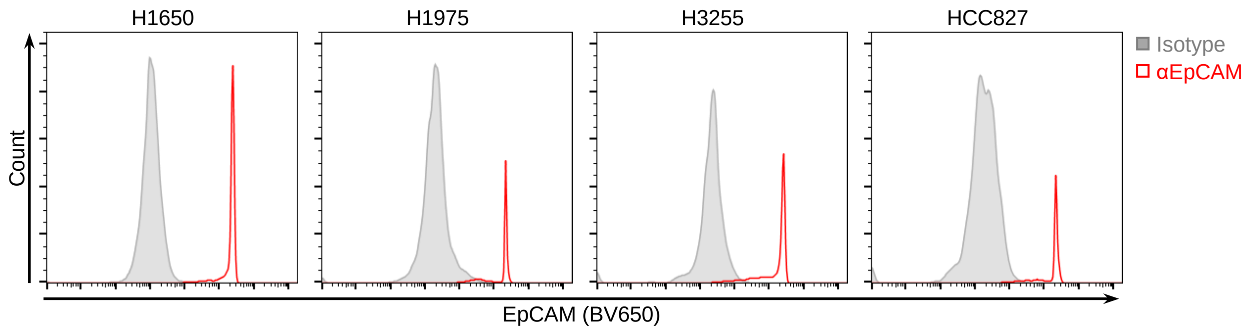


**Figure S3. EpCAM expression in poorly metastatic lung cancer cell lines.** FACS data showing EpCAM expression on poorly metastatic lung cancer cell lines with EGFR mutation, including H1650, H1975, H3255, and HCC827. Date are representative of three independent experiments with similar results.

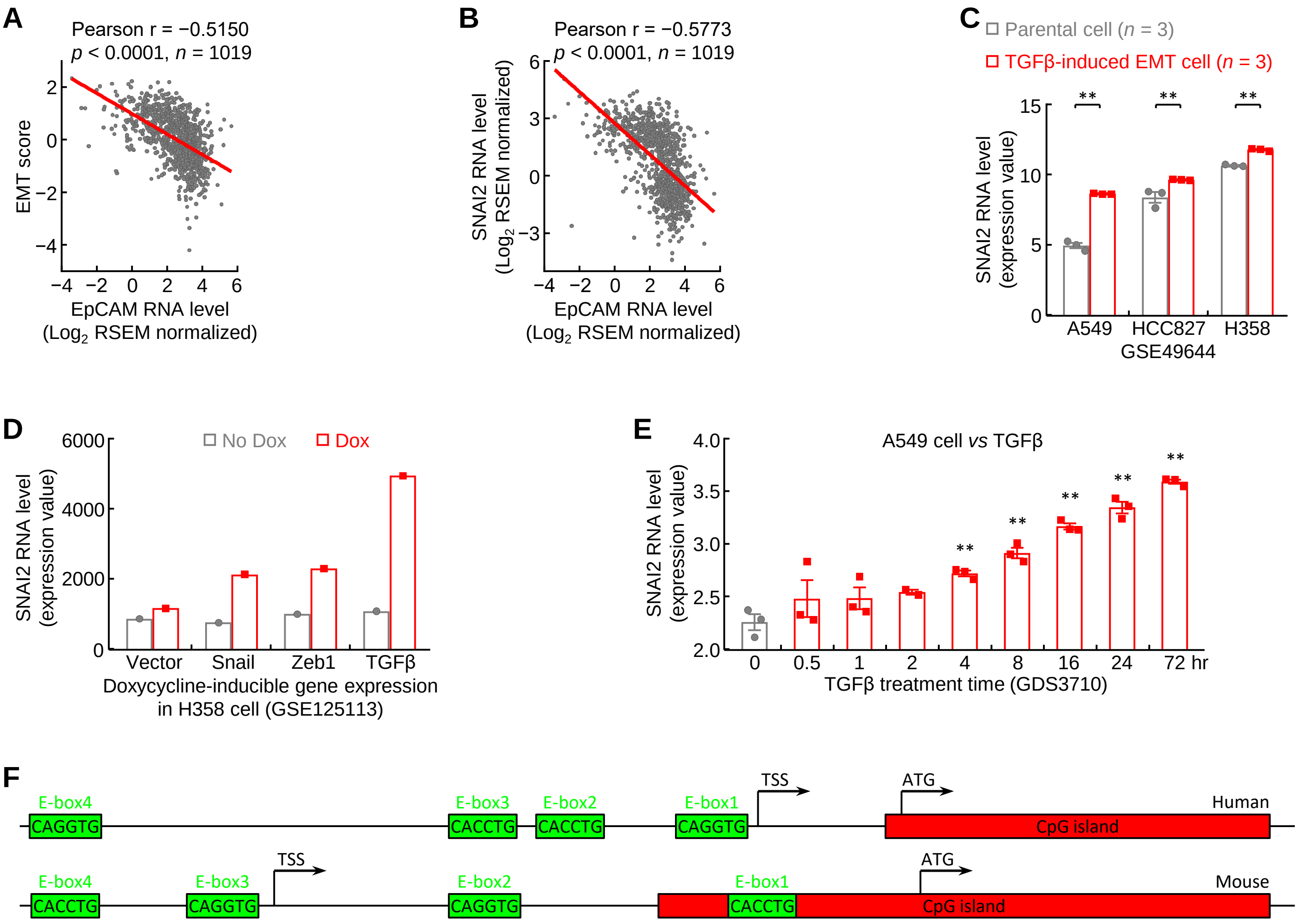


**Figure S4. EMT signaling may contribute to EpCAM repression.** (**A**) TCGA LUNG data showing negative association between EpCAM expression and EMT status. (**B**) TCGA LUNG data showing negative association between EpCAM and SNAI2. (**C**) GSE49644 data showing increased SNAI2 expression in TGFβ-transformed human lung cancer cells. (**D**) GSE125113 data showing increased SNAI2 expression in H358 cell overexpressing TGFβ. (**E**) GDS3710 data showing increased SNAI2 expression by TGFβ in A549 cell. (**F**) Promoter analysis showing four putative SNAI2 binding sites (E-box) and one CpG island in both human and murine *epcam* gene. Student's *t* test (two tailed, unpaired) was performed. Data represent means ± SEM. $**P < 0.01$.

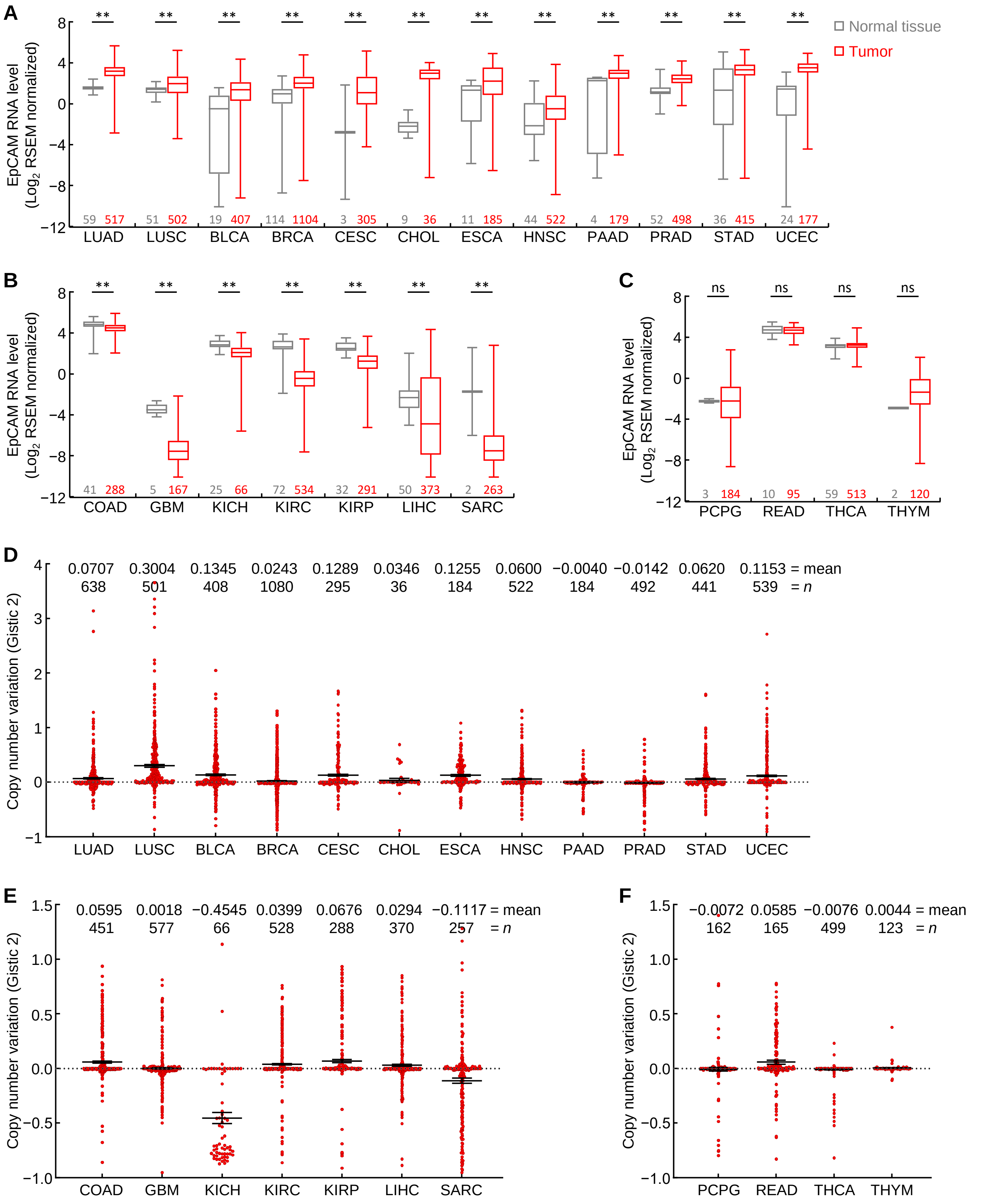


**Figure S5. The expression and copy number of EpCAM in human primary cancers.** (**A-C**) TCGA data showing increased (**A**), decreased (**B**) and comparable (**C**) EpCAM RNA expression in human primary tumors (Tumor: red box) compared to normal tissues (NL: grey box). Patient numbers are indicated below. (**D-F**) TCGA data showing copy number variation of *epcam* gene in human primary tumors. Mean value and patient numbers are indicated above. Student's *t* test (two tailed, unpaired) was performed. Data represent as Box and Whiskers (min to max). $^{**}P < 0.01$; ns, not statistically different.

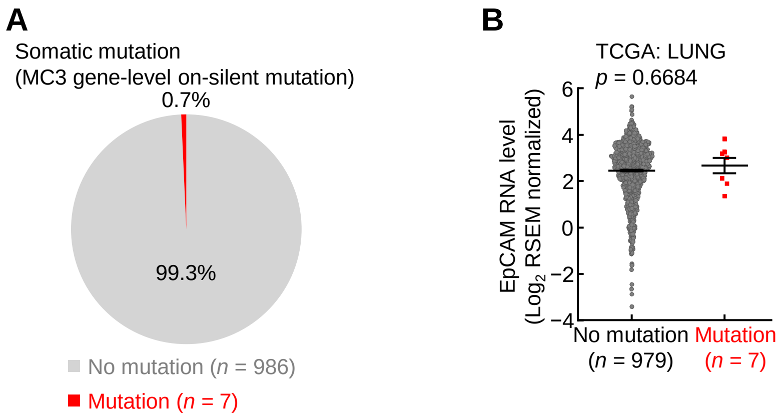


**Figure S6. Gene mutation has minimal impact on EpCAM expression in human primary lung cancer.** (**A**) TCGA somatic mutation data showing low mutation rate of *epcam* in human primary lung cancer. (**B**) TCGA data showing comparable EpCAM RNA expression in *epcam* WT and Mutation groups in human primary tumors. Student's *t* test (two tailed, unpaired) was performed. Data represent means ± SEM. ns, not statistically different.

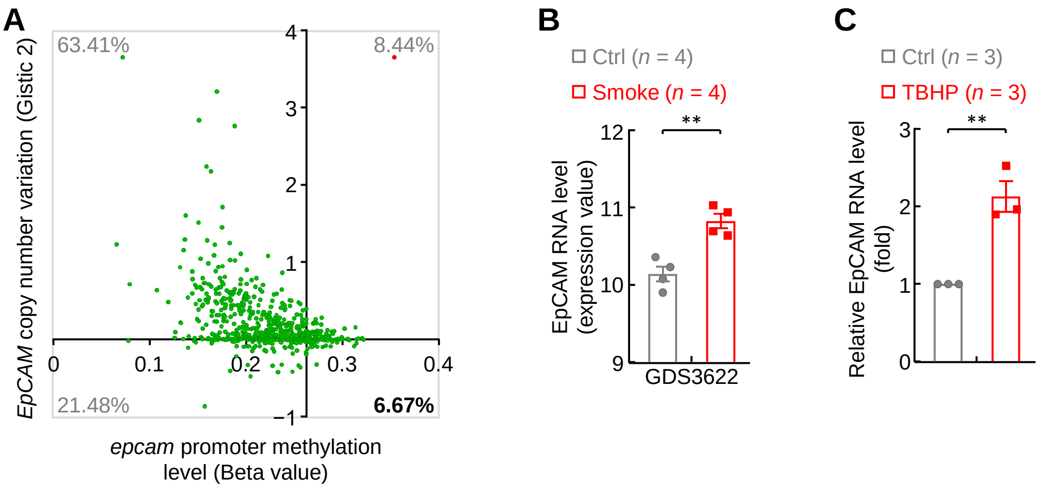


**Figure S7. Smoking induces ROS to upregulate EpCAM expression.** (**A**) TCGA LUNG data showing about 6.6% of lung cancer with low gene amplification and high promoter methylation in EpCAM-upregulated patients. Axis x and y cross at mean value (0.26, 0) of EpCAM promoter methylation level and copy number variation in normal lung. (**B**) GDS3622 data showing increased EpCAM RNA expression in lung tissue by smoking. (**C**) qPCR data showing increased EpCAM RNA expression in TBHP-treated cancer cells. Date are representative of three independent experiments with similar results (**C**). Student's *t* test (two tailed, unpaired) was performed. Data represent means ± SEM. $^{**}P < 0.01$.

| Table S1. Antibody list | | | | | |
|---|---|---|---|---|---|
| **Antibody** | **Clone** | **Cat. No.** | **Company** | **Usage** | **Purpose** |
| Anti-mouse CD16/CD32 | 93, Rat IgG2a, λ | 14-0161-85 | Thermo Fisher Scientific, Waltham,MA, USA | 1.0 µl per sample | FACS |
| Anti-mouse CD45 FITC | 30-F11, Rat IgG2b, κ | 103107 | Biolegend, San Diego, CA, USA | 0.5 µl per sample | FACS |
| Anti-mouse EpCAM PE | G8.8, Rat IgG2a, κ | 12-5791-82 | Thermo Fisher Scientific, Waltham,MA, USA | 0.625 µl per sample | FACS |
| Rat IgG2a κ Isotype Control, PE | eBR2a, Rat IgG2a, κ | 12-4321-80 | Thermo Fisher Scientific, Waltham,MA, USA | 0.625 µl per sample | FACS |
| Human TruStain FcX (Fc Receptor Blocking Solution) | | 422302 | Biolegend, San Diego, CA, USA | 5 µl per sample | FACS |
| Anti-human CD45 AF700 | HI30, Mouse IgG1, κ | 304024 | Biolegend, San Diego, CA, USA | 2 µl per sample | FACS |
| Anti-human EpCAM BV650 | 9C4, Mouse IgG2b, κ | 324226 | Biolegend, San Diego, CA, USA | 5 µl per sample | FACS |
| Mouse IgG2b, κ Isotype Contrl, BV650 | MPC-11, Mouse IgG2b, κ | 400351 | Biolegend, San Diego, CA, USA | 5 µl per sample | FACS |
| Anti-EpCAM | Rabbit polyclonal IgG | PA5-19832 | Thermo Fisher Scientific, Waltham,MA, USA | 1:1000 | IB |
| Anti-GAPDH | 14C10, rabbit mAb | 2118 | Cell Signaling Technology, Danvers,MA, USA | 1:1000 | IB |
| Goat anti-Rabbit HRP | | sc-2054 | Santa Cruz Biotechnology, Dallas, TX,USA | 1:5000 | IB |

| Table S2. Primer list | | | | | |
|---|---|---|---|---|---|
| **Gene** | **Species** | **Accession number** | **Forward (5' to 3')** | **Reverse (5' to 3')** | **Purpose** |
| *gapdh* | human | NM_002046.3 | CCGAGCCACATCGCTCAGACAC | GTGACCAGGCGCCCAATACGAC | RT-PCR |
| *gapdh* | mouse | NM_008084.2 | AGTGCCAGCCTCGTCCCGTA | CAGGCGCCCAATACGGCCAA | RT-PCR |
| *epcam* | human | NM_002354.3 | ACTGGATCATCATTGAACTA | GCCACATCAGCTATGTCCAC | RT-PCR |
| *epcam* | mouse | NM_008532.2 | | | |
